# Bothersome Hematospermia Following Stereotactic Body Radiation Therapy for Prostate Cancer

**DOI:** 10.3389/fonc.2021.765171

**Published:** 2021-11-25

**Authors:** Sarthak Shah, Tamir Sholklapper, Michael Creswell, Abigail Pepin, Jonathan Cantalino, Ryan Andrew Hankins, Simeng Suy, Sean P. Collins

**Affiliations:** ^1^ Department of Radiation Medicine, Georgetown University Hospital, Washington, DC, United States; ^2^ Department of Radiation Medicine, University of Pennsylvania, Philadelphia, PA, United States; ^3^ Department of Urology, Georgetown University Hospital, Washington, DC, United States

**Keywords:** prostate cancer, SBRT (stereotactic body radiation therapy), CyberKnife, hematospermia, 5-alpha reductase inhibitors

## Abstract

**Background:**

Hematospermia following prostate radiation therapy is a benign and often self-limiting side effect. However, it may be bothersome to some men and their partners with a negative impact on sexual quality of life (QOL). This study sought to evaluate the incidence, duration, and resolution of hematospermia in patients following stereotactic body radiation therapy (SBRT) for prostate cancer.

**Methods:**

227 patients treated with SBRT from 2013 to 2019 at Georgetown University Hospital for localized prostate carcinoma with a minimum follow up of two years were included in this retrospective review of data that was prospectively collected. Patients who were greater than 70 years old and/or received hormonal therapy were excluded. Hematospermia was defined as bright red blood in the ejaculate. Time points for data collection included initial consultation, pre-treatment, 1-, 3-, 6-, 9-, 12-, 18-, 24-month. All patients were treated with the CyberKnife Radiosurgical System (Accuray). Data on hematospermia including duration, resolution and recurrence was collected. Utilization of 5-alpha reductase inhibitors was documented at each visit.

**Results:**

227 patients (45 low-, 177 intermediate-, and 5 high-risk according to the D’Amico classification) at a median age of 65 years (range 47-70) received SBRT for their localized prostate cancer. The 2-year cumulative incidence of hematospermia was 5.6%(14 patients). For these patients, all but one patient (93%) saw resolution of their hematospermia by two years post-SBRT. The median time for hematospermia was 9 months post-treatment. Of the 14 patients who reported hematospermia, 70% were managed with 5-alpha reductase inhibitors. Hematospermia was transient in most patients with 70% of the men reporting resolution by the next follow-up visit.

**Conclusion:**

The incidence of bothersome hematospermia following SBRT was low. Hematospermia, as noted by other studies, often self-resolves. 5-alpha reductase inhibitors may lead to quicker resolution of bothersome hematospermia.

## Background

Hematospermia, defined as gross blood in the ejaculate (1), is an uncommon condition in the elderly population (< 1%) (2, 3). It is bothersome to men and their partners but is generally a benign finding that resolves on its own. Standard management is reassurance (1, 4). The most common etiology of hematospermia in the elderly population is iatrogenic including prostate biopsy (5) and prostatic fiducial placement (6). Hematospermia generally resolves in days to weeks (5). The blood is commonly bright red immediately post-procedure but can appear brown in color for months to years after the procedure as prostatic hematomas slowly resolve.

**Figure 1:**
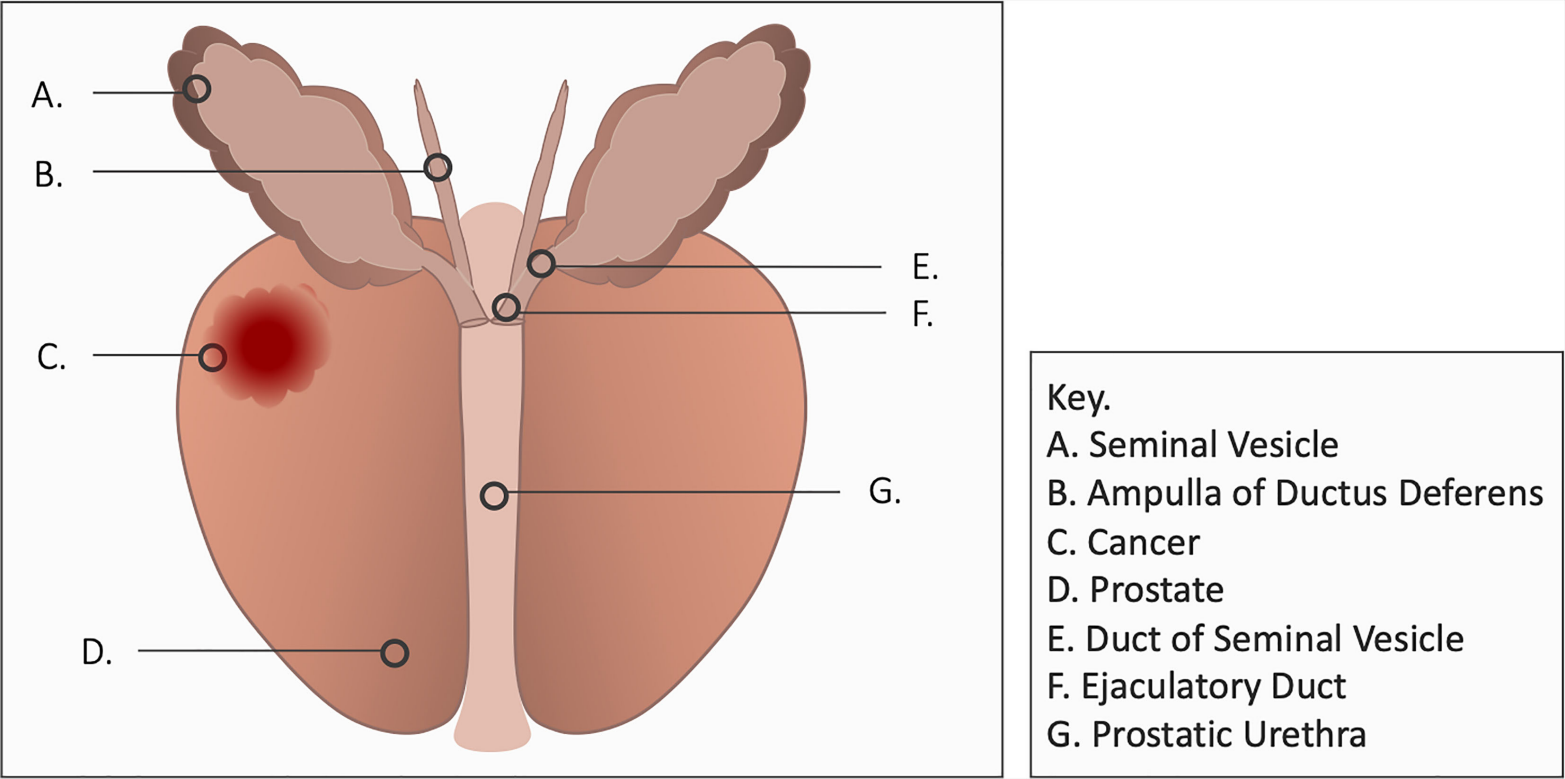
Ejaculatory Ducts of the Prostate.

Bothersome ejaculatory symptoms following prostatic irradiation include reduced fluid volume, ejaculatory pain and hematospermia (7, 8). The etiology of post-radiation hematospermia is unclear but may involve inflammation of the seminal vesicles, vas deferens or ejaculatory ducts (**Figure 1**). It occurs months to years after treatment and generally resolves on its own without interventions but may persist in some men (7, 8). Up to 25% of patients report it following prostate EBRT and/or brachytherapy (7, 8). 5-alpha reductase inhibitors are an effective treatment for hematospermia (9) but they may cause duration-dependent decreased libido (10). This study sought to evaluate the incidence, duration, and resolution of hematospermia in patients following stereotactic body radiation therapy (SBRT) for prostate cancer.

## Methods

### Patient Selection

Patients eligible for this study were those who had histologically-confirmed prostate cancer who were capable of ejaculating. Patients who were greater than 70 years old or received hormonal therapy were excluded from this study due to their known adverse effects on ejaculation (11, 12). Institutional IRB approval was obtained for retrospective review of patient medical records.

### SBRT Treatment Planning and Delivery

SBRT treatment planning and delivery were conducted as previously described (13). Briefly, 4-6 gold fiducials were placed into the prostate *via* a transrectal or transperineal approach. One to two weeks after fiducial placement, CT and MR images were obtained and fused for treatment planning. The clinical target volume (CTV) included the prostate and the proximal seminal vesicles. The planning target volume (PTV) equaled the CTV expanded 3 mm posteriorly and 5 mm in all other dimensions. The prescription dose was 35-36.25 Gy to the PTV delivered in five fractions of 7-7.25 Gy over one to two weeks. In general, men initiated treatment 2-4 weeks following the treatment planning scans.

### Follow-up and Statistical Analysis

Hematospermia was defined as bright red blood in the ejaculate. Brown blood in ejaculate was excluded due to its known association with post-biopsy hematomas (5, 14, 15). Patients were evaluated at initial consultation, the first day of treatment and during routine follow-up visits at one month, every 3 months for the first year and every six months for the second year. Data collected on hematospermia including duration, resolution and recurrence was collected. Time to hematospermia was recorded as the follow-up visit month at which hematospermia was first noted. Duration of hematospermia is calculated as length of time from when hematospermia was noted to subsequent visit when hematospermia was resolved. Utilization of 5-alpha reductase inhibitors was documented at baseline and at all follow-ups.

Analysis of individual characteristics was performed *via* bivariate comparison between patients experiencing hematospermia during the 2-year time and those without hematospermia. Binominal logistic regression was performed for all continuous variables and values were presented as average with standard deviation. Fisher’s exact test was performed for categorical variables and values presented as number experiencing with percent of total cohort. All tests were two-tailed, and a *p* value <0.05 was considered significant. JMP^®^ PRO version 15.0.0 for Macintosh was used to perform the statistical analyses (16).

## Results

227 patients on a prospective quality of life study (IRB#: 2009-510) with baseline ejaculatory capacity treated with prostate SBRT at Georgetown University Hospital from 2013 to 2019 were included in this analysis (**Table 1**). They were ethnically diverse with a median age of 65 years (interquartile range, 62-68 years). The median pre-treatment total serum testosterone level was 373 ng/dL (interquartile range, 287 - 483 ng/dL). When stratified by D’Amico risk group, 45 patients were low-, 179 intermediate-, and 5 high-risk (**Table 1**). For treatment, 90% of patients received 36.25 Gy in five 7.25 Gy fractions. The minimum length of follow-up was 2 years and no patient initiated androgen deprivation therapy prior to SBRT or in the first two years following radiation therapy.

**Table 1:** Baseline patient, disease, and treatment characteristics.

	N	(%)
Age, years		(61.6, 68.0)
Median (IQR)	65.4	(47.2, 70.8)
Mean (range)	64.2	
BMI, kg/m^2^		(25.8, 31.2)
Median (IQR)	28.2	(0.9%)
<18.5	2	(17.1%)
18.5-24.9	37	(47.7%)
25-29.9	103	(31.0%)
30-39.9	67	(3.2%)
>40	7	
Race/Ethnicity		(60.2%)
White or Caucasian	136	(35.0%)
Black of AA	79	(1.3%)
Hispanic	3	(3.5%)
Other	8	
Prostate		
<40	131	(57.7%)
40-60	65	(28.6%)
>60	31	(13.7%)
α1 receptor antagonist		
Yes	197	(88.3%)
No	26	(11.7%)
PDES inhibitor		
Yes	49	(21.7%)
No	177	(78.3%)
Anticoagulant		
Yes	28	(12.3%)
No	90	(17.3%)
Missing	109	(48.0%)
Androgen deprivation therapy		
Yes	6	(2.7%)
No	216	(97.3%)
Testosterone, ng/dL		
Median (IQR)	373	(287, 483)
T-stage		
T1c-T2a	201	(88.5%)
T2b-c	26	(11.5%)
Grade group (Gleason)		
1 (3 +3)	66	(29.3%)
2 (3 + 4)	105	(46.7%)
3 (4 + 3)	51	(22.7%)
4 (4 + 4)	3	(1.3%)
Risk group, D'Amico		
Low	45	(19.8%)
Intermediate	177	(78.0%)
High	5	(2.2%)
Pretreatment PSA, ng/mL		
Median (IQR)	7	(5.3, 10.4)
<10	167	(73.6%)
10-20	49	(21.6%)
>20	11	(4.8%)
SBRT Dose (Gy)		
35	200	(89.7%)
36.25	23	(10.3%)

**Figure 2:**
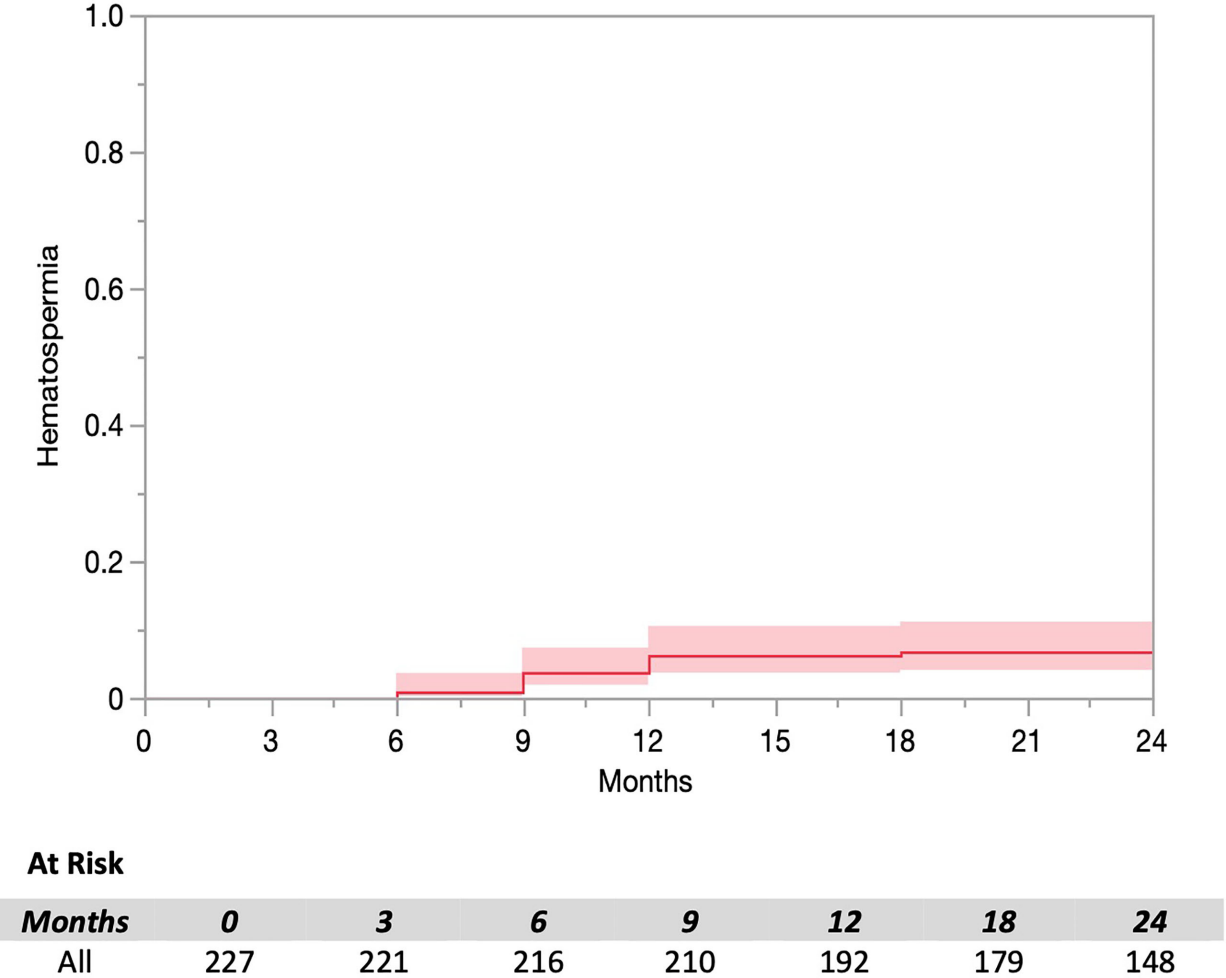
Cumulative incidence of hematospermia following SBRT.

**Figure 3:**
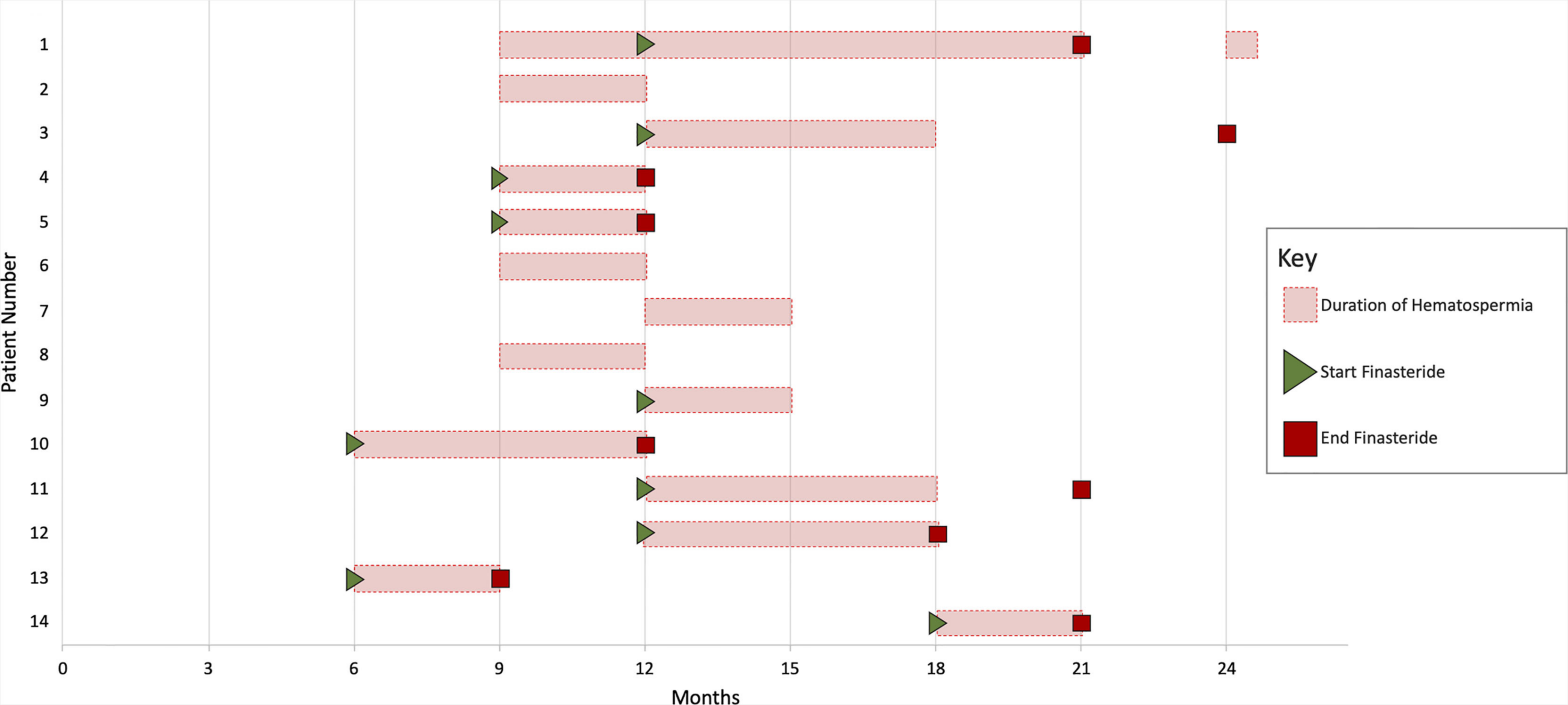
Swimmer’s plot of 14 patients who experienced hematospermia. Error bars represent the beginning and end of hematospermia in months post-SBRT. Green triangles and red boxes symbolize the start and stop of 5-alpha reductase inhibitor treatment respectively.

The prevalence of hematospermia prior to and after SBRT treatment is shown in **Table 2**. At the time of the initial SBRT treatment, no patient reported hematospermia. Levels of patient reported hematospermia increased significantly following treatment (**Table 2**), with 3% of patients reporting blood in the ejaculate at 3 and 6 months post-SBRT (*p* < 0.0001). While a low level of hematospermia was seen throughout the second years of follow-up, our 24-month prevalence of hematospermia was approaching baseline values (**Table 2**). The overall cumulative incidence of hematospermia two years post-SBRT was 5.6% (14 patients) (**Figure 2**). The mean time to developing hematospermia was 9 months post-SBRT. 70% of the patients were treated with 5-alpha reductase inhibitors. The mean duration was 3 months (range 3-12 months). **Figure 3** depicts a Swimmer’s plot of hematospermia prevalence and 5-alpha reductase inhibitor utilization.

**Table 2:** Hematospermia Incidence after SBRT.

				Men with hematospermia							
	Total	No prior hematospermia		Overall		Receiving Finasteride				Cumulative clearance of hematospermia	
	n	n	(%)	n	(%)	Yes	(%)	No	(%)	n	(%)
Treatment start	226	226	(100.0%)	0	(0.0%)	0		0		0	
1month	224	224	(100.0%)	0	(0.0%)	0		0		0	
3 months	213	213	(100.0%)	0	(0.0%)	0		0		0	
6 months	208	206	(99.0%)	2	(1.0%)	2	(100.0%)	0	(0.0%)	0	(0.0%)
9 months	202	195	(96.5%)	6	(3.0%)	2	(33.3%)	4	(66.7%)	1	(14.3%)
12 months	193	180	(93.3%)	6	(3.1%)	3	(50.0%)	3	(50.0%)	7	(53.8%)
15 months	176	168	(95.5%)	1	(0.6%)	1	(100.0%)	0	(0.0%)	7	(87.5%)
18 months	185	173	(93.5%)	2	(1.1%)	2	(100.0%)	0	(0.0%)	10	(83.3%)
21months	157	146	(93.0%)	0	(0.0%)	0		0		11	(100.0%)
24 months	159	148	(93.1%)	1	(0.6%)	0	(0.0%)	1	(100.0%)	10	(90.9%)

## Discussion

To our knowledge, this is the first study to report the hematospermia incidence following prostate SBRT. Hematospermia was uncommon at any time point and transient in most cases. The prevalence of hematospermia peaked at 6-9 months. From our clinical experience, hematospermia was rare greater than two years post-SBRT. These results appear similar to brachytherapy (7, 8). Hematospermia is a known complication of conventionally fractionated IMRT (1), however we could not identify evidence for the incidence in the current literature. Future work should compare the incidence of hematospermia following conventionally fractionated IMRT and SBRT.

Post-SBRT hematospermia is likely secondary ejaculatory duct inflammation (1, 4). Inflammation of the ejaculatory apparatus is a common cause of hematospermia (1, 4). Etiologies include epididymitis, urethritis, prostatitis and seminal vesiculitis. The timing of hematospermia following SBRT is similar to the phenomena of late urinary symptom flare (17). Late urinary symptom flare is a transient increase in urinary symptoms seen several months following SBRT (17). It resolves on its own with time with a percentage of patients requiring a short course of anti-inflammatory medications (17). The exact etiology is unknown but likely involves post-RT inflammation of the bladder neck/prostatic urethra (18, 19).

5-alpha reductase inhibitors reduce hematospermia by reducing blood flow to the prostate (9). The Optimal Length of finasteride treatment is unknown. In general, we prescribe for three to six months then discontinued due to adverse sexual side effects. Hematospermia recurred in 1 patient but responded to a second course of 5-alpha reductase inhibitors (**Figure 3**).

### Limitations

This study had several limitations. The true incidence of hematospermia is difficult to know because many elderly men are not highly sexually active. In addition, men do not commonly examine their ejaculate and if they did, they may not be able to distinguish bright red blood from brown blood. When assessing treatment toxicity, we did not specifically ask about hematospermia. In addition, it is impossible to know if hematospermia is secondary to radiation or fiducial placement. Men likely only reported hematospermia to their physician when bothersome to them and/or their partner (20, 21). Currently, there is no validated questionnaire to examine hematospermia (22). The cumulative incidence might have been higher if men would have been asked specifically about hematospermia at the time of follow-up and/or were able to privately document their experience *via* questionnaire (7, 23). In addition, brown blood was not recorded in our medical records do to its known association with episodic resolution of post-biopsy hematomas (24).

## Conclusions

Hematospermia is a bothersome self-limiting symptom experienced by a small percentage of men following prostate SBRT. 5-alpha reductase inhibitors may lead to quicker resolution of bothersome hematospermia.

## Data Availability Statement

As per the wishes of the patients, the patient dataset is only available to those conducting research at the Georgetown University Medical Center. Requests to access the datasets should be directed to SPC9@gunet.georgetown.edu.

## Ethics Statement

The studies involving human participants were reviewed and approved by Georgetown University Institutional Review Board. The patients/participants provided their written informed consent to participate in this study.

## Author Contributions

SaS was the lead author and participated in data collection and manuscript revision. TS and MC also contributed equally to SiS for this work and participated in data analysis, manuscript drafting, table/figure creation, and manuscript revision. AP, JC, BC, and RH aided in review and revision of the manuscript. SS is a senior author who organized the data and participated in its analysis. SC was the principal investigator who initially developed the concept of the study and the design, aided in data collected, and drafted and revised the manuscript. All authors contributed to the article and approved the submitted version.

## Funding

This work was supported by The James and Theodore Pedas Family Foundation. The Department of Radiation Medicine at Georgetown University Hospital receives a grant from Accuray to support a research coordinator. We gratefully acknowledge the grant R01MD012767 from the National Institute on Minority Health and Health Disparities (NIMHD), NIH to SC.

## Conflict of Interest

SC serves as clinical consultants to Accuray Inc.

The remaining authors declare that the research was conducted in the absence of any commercial or financial relationships that could be construed as a potential conflict of interest.

## Publisher’s Note

All claims expressed in this article are solely those of the authors and do not necessarily represent those of their affiliated organizations, or those of the publisher, the editors and the reviewers. Any product that may be evaluated in this article, or claim that may be made by its manufacturer, is not guaranteed or endorsed by the publisher.

## Abbreviations

ADT, androgen deprivation therapy; CTV, clinical target volume; DVH, dose-volume histogram; GTV, gross target volume; PTV, planning target volume; QoL, quality of life; SHIM, Sexual Health Inventory for Men; EBRT, external beam radiation therapy; SBRT, stereotactic body radiation therapy; EPIC, Expanded Prostate Index Composite.
